# Supplementary material for: HIVprotI: an integrated web based platform for prediction and design of HIV proteins inhibitors
Source: J Cheminform. 2018 Mar 9;10:12. doi: 10.1186/s13321-018-0266-y (PMC5845081; doi:10.1186/s13321-018-0266-y)
Supplement: Supplementary file 2 — Additional file 2. Source code of HIVProtI web server. [file 13321_2018_266_MOESM2_ESM.zip › HIVprotI_Source-code/hivprotI/weka-3-6-6/doc/weka/clusterers/NumberOfClustersRequestable.html]

NumberOfClustersRequestable


---


|  |  |  |
| --- | --- | --- |
| **Overview** \| **Package** \| **Class** \| **Tree** \| **Deprecated** \| **Index** \| **Help** \| **Weka's home** | |  |
| **PREV CLASS**   **NEXT CLASS** | **FRAMES**    **NO FRAMES**     **All Classes** |  |
| SUMMARY: NESTED \| FIELD \| CONSTR \| METHOD | DETAIL: FIELD \| CONSTR \| METHOD |  |


---


## weka.clusterers Interface NumberOfClustersRequestable

**All Known Implementing Classes:**: EM, MakeDensityBasedClusterer, SimpleKMeans

---

``` public interface NumberOfClustersRequestable ```

Interface to a clusterer that can generate a requested number of
clusters

**Version:**
:   $Revision: 1.2 $

**Author:**
:   Mark Hall

---

| **Method Summary** | |
| --- | --- |
| `void` | `setNumClusters(int numClusters)`             Set the number of clusters to generate |

**Method Detail**

### setNumClusters

```
void setNumClusters(int numClusters)
                    throws java.lang.Exception
```

:   Set the number of clusters to generate

    :   **Parameters:**: `numClusters` - the number of clusters to generate **Throws:**: `java.lang.Exception` - if the requested number of clusters in inapropriate


---


|  |  |  |
| --- | --- | --- |
| **Overview** \| **Package** \| **Class** \| **Tree** \| **Deprecated** \| **Index** \| **Help** \| **Weka's home** | |  |
| **PREV CLASS**   **NEXT CLASS** | **FRAMES**    **NO FRAMES**     **All Classes** |  |
| SUMMARY: NESTED \| FIELD \| CONSTR \| METHOD | DETAIL: FIELD \| CONSTR \| METHOD |  |


---
